# The Interplay Between Status and Affection Needs: Testing the Imbalanced Needs Theory of Aggression in Adulthood

**DOI:** 10.1177/08862605221084741

**Published:** 2022-03-26

**Authors:** Jelle J. Sijtsema, Siegwart M. Lindenberg

**Affiliations:** 1 Developmental Psychology, 7899 Tilburg University, the Netherlands; 2 Sociology, 3647 University of Groningen, The Netherlands; 3 Social Psychology, 7899 Tilburg University, The Netherlands

**Keywords:** social goals, aggression, development, status, affection

## Abstract

Status and affection are both goals related to social needs. The *imbalanced needs theory of aggression* proposes that although aggression can be used to realize status, this strategy is detrimental for realizing affection in the same social context. Thus, to the degree that the social circles overlap in which status and affection needs are realized, it becomes more costly (in terms of affection) to achieve status via aggression. This theory was tested for different forms of aggression, in different contexts, in a sample of adults from the general population (*N* = 253, M age = 29.95, SD = 2.60, 78% female). Participants reported on social needs with the Interpersonal Goals Inventory and reported on general measures of physical and social aggression, as well as rule breaking, and aggression at the workplace and in intimate partner relationships. As hypothesized, status needs were associated with physical aggression when affection needs were weak. This interaction, though to a lesser degree, also extended to social forms of aggression and rule breaking. At the workplace, aggression was only related to weak affection needs, whereas aggression in intimate partner relationships was, as expected, unrelated to both social needs. Together, these findings support the results of an earlier test of the *imbalanced needs theory of aggression* in adolescence, and encourage more research into the link between aggression and the satisfaction of social needs.

Interpersonal aggressive behavior is often explained by individual characteristics related to personality traits, psychopathology, and associated factors (Anderson & Bushman, 2002; Babcock et al., 2014). However, what are the most important *social* determinants of interpersonal aggression? Decades of research into this question have identified several social determinants of aggression, such as social rejection, violent environments, and violent media (Warburton & Anderson, 2015). Thus, socially determined aggression is mostly seen as a reaction to a hostile environment. We have no reason to doubt the research in this area. However, much aggression also occurs without exposure to hostile environments, in the pursuit of daily life, and a pressing question is what the determinants of this kind of aggression are, if it is not due to psychopathology or hostile environments. Recently, we suggested that one needs to look at the social function of aggression from the perspective of a need-based goal framework, and we found empirical support for such an approach in adolescence (Sijtsema et al., 2020). The question remains whether we also find support for this theory for adults.

## The Imbalanced Needs Theory of Aggression

The background of the “imbalanced needs theory of aggression” is that social goals are important determinants of behavior (Lindenberg, 2013) and have been increasingly explored explicitly in research on the behavior of children and adolescents (Crick & Dodge, 1994; Heidgerken et al., 2004; Ojanen et al., 2005; Renshaw & Asher, 1983; Sijtsema et al., 2009; Volk et al., 2012). The main focus of these studies was on two kinds of goals that are often thought to be negatively correlated, but that turned out to be orthogonal (Ojanen et al., 2005): a communal goal, related to warmth, support, love and intimacy, also referred to as *affection goals*, and an agentic goal, related to power, dominance, and a feeling of superiority, also referred to as *status goals* (see also Locke, 2003). Both kinds of goals can be linked to universal interpersonal needs (Anderson et al., 2015; Bakan, 1966; Lindenberg, 2013). Most individuals endorse both status and affection goals, to differing degrees (Ormel et al., 1997; Steverink et al., 2020). Attaching importance to status goals has been associated with aggression (Caravita & Cillessen, 2012; Ojanen & Findley-Van Nostrand, 2014; Reijntjes et al., 2016; Sijtsema et al., 2009). However, aggression is likely to reduce realizing affection in the relationship with the very same others one would like to be superior to. This very fact led us to formulate and test what we called the *imbalanced needs theory of aggression* among adolescents (Sijtsema et al., 2020). This theory of social determinants of aggression rests on two main theoretical pillars: first, individuals have social needs, for status and for affection, to varying degrees. Second, aggression is an effective means for achieving status as long as it does not diminish the realization of affection. It follows that the strength of the status goal will be positively correlated with showing aggression when status and affection can be realized in separate social circles. To the degree that these circles overlap, two things happen, compared to separate circles: (a) using aggression as a means for achieving status will become costlier in terms of losing affection; and (b) because using aggression for achieving status is detrimental for the satisfaction of social needs, using aggression as a means for achieving status will become more socially disapproved when these circles overlap. As a result, the correlation of the strength of the status goal and aggression will be lower, except for people who happen to be high on the status goal and low on the affection goal. The theory also predicts an age effect: compared to childhood and early adolescence, social circles in later adolescence will increasingly overlap, and thus the correlation between the strength of the status goal and the use of aggression is predicted to decline during later adolescence. In adulthood, this correlation is predicted to decline even further, because the social circles for the realization of status and affection are even more integrated (Snijders et al., 2013) and social disapproval of showing aggression becomes even stronger (e.g., Laub & Sampson, 1993), because “being adult” becomes associated with the expectation of being able to restrain one’s aggression (Sweeten et al., 2013). Negative reputational effects of showing aggression (as is observed with gossiping; see Feinberg et al., 2012) will reinforce this expectation. Conversely, self-regulation needed to meet these expectations is likely to become stronger with age, allowing greater restraint on the use of aggression when it interferes with other important goals and external regulation (Williams et al., 1999).

The theory also implies context effects in addition to the overlap of social circles: (a) if the context provides good alternatives for achieving status without the use of aggression, the correlation between the strength of the status goal and aggression will be weak, compared to a situation without this alternative. (b) If the context makes the achievement of affection more important than the achievement of status, then the correlation between the strength of the status goal and aggression will be also be weak.

In our previous study on the *imbalanced needs theory of aggression* (Sijtsema et al., 2020), we found that the strength of the status goal was associated with direct aggression across adolescence. However, in middle adolescence, this association was only observed when the affection goal was weak. We argued that these findings were due to more integrated social circles of interaction with age. That is, in comparison to younger adolescents, status and affection for older adolescents were realized more often in the same circle of interaction, namely the peer group. Thus, as the circles of interaction for the achievement of status and affection overlap more, aggression will become more costly and decline. The theory predicts that in adulthood, the circles for the realization of status and affection overlap even more than in later adolescence and that the use of aggression for the achievement of status meets with even greater disapproval than in later adolescence. We will apply this theory to different forms of aggression and to different social contexts and test the hypotheses that derive from these applications.

**Status and different forms of aggression.** In line with our previous test of the theory for adolescence regarding physical aggression, our first hypothesis for adults to be tested relates to physical aggression as well. *Physical aggression hypothesis*: In adulthood, the strength of the status goal is related to physical aggression, but only when the affection goal is weak.

There are also other forms of aggression, especially social aggression and rule breaking, and the question arises how the theory applies to these forms. Social aggression refers to the intention to harm somebody’s social standing and reputation (e.g., “to ridicule someone behind their back”), rather than harming somebody physically. Because social aggression has been shown to be a means for the achievement of status (Adler & Adler, 1998; Prinstein & Cillessen, 2003), just like physical aggression, our second hypothesis to be tested relates to social aggression in analogy to the hypothesis about physical aggression. *Social aggression hypothesis:* In adulthood, the strength of the status goal is related to social aggression, but only when the affection goal is weak.

Theories that aim to explain the mechanism behind a phenomenon should not just explain under what conditions the phenomenon occurs, but also under what conditions it does not occur. Rule-breaking as a form of aggressive behavior may be used for achieving status only in adolescence. The reason for this is that rule breaking has been linked to the maturity gap—the discrepancy between social and biological maturity—as a form of antisocial behavior, including aggression, that proves status in the form of one’s autonomy vis-a-vis parents and other authorities (Dijkstra et al., 2015; Koepke & Denissen, 2012). The maturity gap closes progressively in later adolescence (Moffitt, 1993), which makes that the circles for the realization of status and affection become even more integrated and that the use of aggression for any goal achievement (including status) becomes socially less acceptable (Weaver & McNeill, 2015). Our third hypothesis to be tested thus concerns rule breaking. *Rule-breaking hypothesis*: in adulthood, the strengths of the status goal is unrelated to rule breaking, irrespective of the strength of the affection goal.

**Status and aggression in different contexts.** We extend the application of our theory to two specific contexts that are relevant for virtually everybody: the workplace and intimate relationships. In these specific contexts, special circumstances might make aggression more or less independent of the strength of the status goal. The imbalanced needs theory of aggression also implies that imbalance may be avoided when aggression becomes unrelated to need fulfillment. This is the case (a) when, in particular contexts, status can be better achieved through other means than aggression, or (b) affection is much more relevant in this context than status. We therefore also investigated aggression in the work place (where there are other avenues for status achievement) and aggression in intimate relationships (where affection is presumably much more important than status).

With regard to the form of aggression, we opted for behaviors that pertain specifically to these contexts. For the work place, we focused on “work place aggression”, that is, on more indirect forms pertaining to social aggression, rather than physical forms of aggression; and for intimate partner relationships, we focused on the most frequently observed forms of intimate partner aggression, that is, psychological aggression and negotiation strategies that indicate the presence of conflict (Feldman & Gowen, 1998; Straus et al., 1996).

***Work context*.** In the work context, status is more pronounced than in most other contexts in adulthood. As such, people may adopt different means to achieve status in this context, with aggression being one of them. Previous studies on workplace aggression indicate a relatively high prevalence of workplace aggression and bullying, suggesting that such behaviors are far from uncommon in adulthood (Aquino & Thau, 2009). Several studies also shed light on the motivation for workplace aggression, indicating that aggression can be used as a strategy to rise in the ranks in work settings (Neuman & Baron, 2005; Spector et al., 2006). This idea is supported by a recent empirical account showing that also adults who use aggression can be rewarded in terms of social status (Ruschoff et al., 2015).

However, even though aggression may still be a way to achieve status at the work place, at work there are other avenues for status that run via competence and promotion, for example via valued knowledge, skills, work effort, and via earning respect and positions (Blau, 1964; Cheng et al., 2013; Maner, 2017; Ng et al., 2005). These alternative ways of achieving status may greatly reduce the use of achieving status via displays of dominance (aggression), thereby also reducing the influence of the strength of the status goal on aggression. In short, our fourth hypothesis concerns the work context. W*ork place hypothesis*: in work settings, the strength of the status goal is only weakly related or unrelated to aggression.

***Romantic contexts*.** Social relationships, and especially intimate relationships, are likely to be more stable in adulthood than during adolescence (Zimmer-Gembeck, 2002). As romantic relationships become longer lasting, competitive mating contexts are likely to become less numerous (Collins, 2003; Sassler, 2010). Thus, in adult romantic contexts, affection and intimacy, and not status, are likely to be highly salient. In particular, just as in overlapping social circles, in relationships with a romantic partner, status concerns may impede the realization of affection and thus the quality and stability of the relationship (Sadikaj et al., 2017). It is thus likely that aggression between romantic partners (intimate partner violence) is not socially induced by the status goal, but is due to individual dispositions, such as personality disorders related to antisocial behavior (e.g., Holtzworth-Munroe, 2000; Ross & Babcock, 2009). Therefore, our fifth hypothesis to be tested is about romantic contexts. *Romantic context hypothesis:* for intimate partner relationships, the strength of the status goal is unrelated to aggression, irrespective of the strength of the affection goal.

In the following, we test these hypotheses in a sample of adults from the general population, while accounting for age and sex.

## Method

### Participants and Procedure

Data were collected among 253 Dutch adults from the general population (22% males; *M* age = 29.95, *SD* = 2.60; age range 18–67) as part of a data collection on dominance, antisocial personality traits, and life events. Sixty-eight percent of the participants were highly educated or currently followed a university or college education and 70% had a full-time or part-time job. Data collection was administered by three graduate students who approached participants from their personal networks via e-mail and Facebook. Furthermore, participants were asked to forward the invitation to their network to increase participation. Participants received a letter including information about the study, with a hyperlink to the digital questionnaire. Participants were fully informed about the subject of the study, the anonymous treatment of data, and the option to withdraw from the study at any time. These research procedures are in line with the ethical standards and guidelines in the Netherlands, following the Declaration of Helsinki (1964). In total, 410 individuals were invited to fill out the online questionnaire. Of these, 157 clicked on the link to the study, but did not complete all measures or only filled out their sex and age. A chi-square test showed that dropouts were more likely to be male compared to those who participated (Χ^*2*^ = 7.46, *p* < .01), but did not differ in age. Little’s MCAR test indicated that data were missing completely at random (Χ^*2*^= 17.24, *df* = 29, *p* = .96).

### Instruments

**Aggressive behavior.** Physical aggression, social aggression, and rule breaking were assessed with an adapted version of the Subtypes of Antisocial Behavior (STAB) questionnaire (Burt & Donnellan, 2009). Participants were asked how often they felt or behaved aggressively in the past 12 months on a 5-point scale, ranging from 1 (never) to 5 (always). Physical aggression was assessed with a 7-item subscale (α = 0.79), including items such as “got into physical fights” and “threatened others.” Social aggression was assessed with 10 items (α = 0.78), such as “damaged someone’s reputation on purpose” and “ridiculed someone behind their back.” Rule breaking was assessed with 8 items (α = 0.82), such as “stole things from a shop” and “sold drugs.” Scores were averaged for each subscale across the items with higher scores indicating higher levels of aggression or rule breaking.

**Workplace aggression.** To assess workplace aggression, we adapted the perceived workplace victimization questionnaire (Aquino, 2000). Participants were asked how often they behaved aggressively at the workplace in the past 6 months on a 5-point scale, ranging from 1 (never) to 5 (20 times or more). Workplace aggression perpetration was assessed with five items (α = 0.61), including items such as “gossiped about a coworker” and “cursed at a coworker.” Scores were averaged across the items with higher scores indicating higher levels of aggression.

**Intimate partner violence.** To assess aggression in romantic contexts, we opted for the Revised Conflict Tactics Scale (CTS2; Straus et al., 1996), which is the most commonly used instrument to assess intimate partner violence in clinical and general population samples (Archer, 2004; Johnson, 2008). Participants filled out this instrument when they were currently in a romantic relationship or had been in a romantic relationship in the past year. We included two subscales of this instrument that tapped into psychological aggression and conflict negotiation strategies. Conflicts in intimate relationships are potentially signs of lower affection. The psychological aggression scale consists of eight items and assesses verbal aggression, coercion, and threats. This scale includes items such as, “I accused my partner of being a worthless lover” and “I threatened to hit or throw something at my partner.” Conflict negotiation strategies were assessed with six items that showed some inclination for compromise but were also an indication of having to deal with conflicts, such as “I proposed a compromise to end a fight” or “I explained my side of a disagreement to my partner.” Answers were rated on an 8-point scale ranging from 0 (never) to 7 (more than 20 times) in the past 12 months. Cronbach’s alphas were 0.88 for both psychological aggression and conflict negotiation strategies. Scores were averaged across all items for each subscale, with higher scores indicating more psychological aggression and more use of conflict negotiation strategies, respectively.

**Social goals.** In the current study, we view agentic and communal goals as trait-like interpersonal motivations that in part resemble universal social needs important for human development (Anderson et al., 2015). The status goal (striving to be dominant and assertive) and the affection goal (seeking relational warmth) were assessed with the Interpersonal Goal Inventory in adults (Dryer & Horowitz, 1997; Locke, 2003). Under the frame “When working with someone on a task, it is important to me that…,” participants were asked to rate the subjective importance of 32 interpersonal outcomes on a 7-point scale ranging from 1 (completely disagree) to 7 (completely agree). Items related to agency were averaged to construct the subscale that assessed the strength of the status goal (i.e., a combination of the Agentic and Separate-Agentic scale; 8 items, α = 0.57). Example items are “…Be assertive with the other person” and “… Be firm when I need to be.” Similarly, items related to communion were averaged to construct the subscale that assessed the strength of the affection goal (i.e., a combination of the Communal and Communal-Separate scale; 8 items, α = 0.68). Example items are “…Be supportive of the other person’s goals” and “… Share openly my thoughts and ideas.”

Following existing literature (Locke, 2003), information represented in the eight goal scales was summarized into overarching status and affection goal vector scores in the circumplex space, via the formulas below:

Strength of the status goal = Agentic _ Submissive + [.707 × (Communal and Agentic + Separate and Agentic _ Communal and Submissive _ Separate and Submissive)]

Strength of the affection goal = Communal _ Separate + [.707 × (Communal and Agentic + Communal and Submissive _ Separate and Agentic _ Separate and Submissive)].

These scores were used to assess status and affection goals respectively. Scores on the four intermediate scales (Communal and Agentic, Communal and Submissive, Separate and Agentic, and Separate and Submissive) were multiplied by .707 because this is the cosine of a 45° angle (the angle of those scales, relative to the status and affection goal vectors).

### Data Analysis

We calculated means, standard deviations, and ranges of all study variables. Because age showed a bimodal distribution, it was recoded into a dummy categorizing participants from 18 up to 30 years and those aged 30 years and over into two different groups. Using independent samples *t*-tests, we calculated mean differences on all variables between these age groups. Next, we computed Pearson correlations between all continuous study variables. Finally, we performed linear regression analyses to test our hypotheses regarding the interaction between status and affection goals. Examining the distribution of the outcome measures indicated relatively high levels of skewness and kurtosis with values above 2 or below −2 (George & Mallery, 2010) in physical aggression, rule breaking, and psychological aggression between romantic partners. Formal tests of normality indicated that the distributions of all aggression outcome measures were non-linear (Kolmogorov-Smirnov test statistics between 0.097 and 0.250, *p*s < .05). Therefore, we conducted bootstrapped correlation analyses, creating 1000 random samples to ensure the robustness of our results. This procedure is less sensitive to skewed outcome measures and yields more reliable coefficients and 95% confidence intervals. Next, we tested whether the assumption of a linear relationship between the status goal and the outcome measures was linear. To this end, we produced scatter plots for each outcome measure, which all indicated at least some linearity. We also produced P-P-plots for each regression analysis, which also suggested linearity. Moreover, multicollinearity indices, such as the Variance Inflation Factor (VIF) and the tolerance, indicated no overlap between the independent variable in explaining the various outcome measures, with scores all within the acceptable range (VIFs <1.07; tolerance >0.94). Finally, we calculated scatterplots between the standardized residuals and the standardized predicted values for each regression model, which showed no clear patterns thereby indicating homoscedasticity. To interpret the findings, significant interactions were plotted using simple slope analysis (Aiken & West, 1991). To reduce potential problems with multicollinearity and to ensure that the values plotted in the figures are accurate representations of the data, independent variables were standardized to a mean of 0 and a standard deviation of 1. All analyses were performed in IBM SPSS 24.0 and hypotheses were tested two-sidedly using a *p*-value of <.05 to indicate significance.

## Results

### Descriptive Analyses

In Table 1 and 2, means, standard deviations, and correlations of all study variables are presented for the whole sample (a) and the two age groups separately (b). Independent sample *t-*tests showed that younger adults reported more physical aggression, social aggression, and rule breaking, as well as more psychological aggression and conflict negotiation strategies in intimate partner relationships (*t*s > 3.22, *p*s < .05). Age groups did not differ in social goals and workplace aggression. Correlations further indicated that the strength of the affection goal was negatively associated with social aggression in younger adults, suggesting that those with a stronger affection goal reported less social aggression. In both age groups, status and affection goals were unrelated to the other antisocial outcomes.

**Table 1.** *Means, Standard Deviations, and Correlations of All Study Variables (Complete Sample; N = 253)*.

	1	2	3	4	5	6	7	8	9		
										M	*SD*
1. Age (in years)	—	0.03	0.05	−0.22**	−0.36**	−0.21**	−0.05	−0.21**	−0.22**	29.95	12.60
2. Status goals			−0.03	0.01	0.03	0.01	0.11	0.04	0.05	0.16	1.12
3. Affection goals				−0.03	−0.17**	−0.13*	−0.11	−0.12	0.01	2.30	1.45
4. Physical aggression					0.56**	0.50**	0.11	0.41**	0.23**	1.34	0.38
5. Social aggression						0.66**	0.39**	0.45**	0.27**	1.49	0.38
6. Rule-breaking							0.28**	.34**	0.16*	1.12	0.17
7. Workplace aggression								0.14*	0.18**	1.26	0.28
8. Psychological IPV^a^									0.50**	0.94	1.07
9. Negotiation IPV^a^									−	3.63	1.64

*Note.* IPV = Intimate Partner Violence.

a *n* = 217.

*p < .05, **p < .01.

**Table 2.** *Means, Standard Deviations, and Correlations of All Study Variables (Age 18-30 below and age 31-67 above the diagonal)*.

	1	2	3	4	5	6	7	8	9	Age 18–30 (*n* = 190)		Age 31–67 (*n* = 63)		Independent samples t-tests	
										M	*SD*	M	*SD*	*t*-value	df
1. Age (in years)	—	0.17	0.13	−0.07	−0.10	−0.01	−0.14	−0.17	0.01	23.31	2.74	50.00	8.87	−36.69***	251
2. Status goals	0.03	—	0.01	0.05	−0.01	0.09	0.14	0.12	−0.02	0.17	1.11	0.16	1.17	0.07	251
3. Affection goals	−0.09	−0.04	—	0.01	−0.05	−0.11	−0.03	0.05	−0.02	2.27	1.51	2.42	1.25	−0.74	251
4. Physical aggression	−0.02	−0.00	−0.03	—	0.56**	0.18	0.31*	0.16	0.20	1.39	0.41	1.19	0.21	3.75***	251
5. Social aggression	−0.16*	0.05	−0.19*	0.53**	—	0.64**	0.51**	0.37**	0.42**	1.57	0.39	1.27	0.25	5.88***	251
6. Rule-breaking	−0.06	−0.00	−0.13	0.50**	0.64**	—	0.37**	0.11	0.16	1.14	0.18	1.06	0.08	3.48**	251
7. Workplace aggression	0.07	0.10	−0.13	0.08	0.38**	0.26**	—	0.16	0.27	1.27	0.29	1.24	0.25	0.74	251
8. Psychological IPV	−0.02	0.03	0.01	0.20*	0.19*	0.12	0.17*	—	0.35**	3.83	1.66	3.01	1.41	3.22**	215
9. Negotiation IPV	−0.06	0.07	−0.14	0.41**	0.41**	0.34**	0.09	0.50**	—	1.07	1.06	0.52	1.02	3.31**	215

*p < .05, **p < .01, *** *p* < .001.

*Note.* IPV = Intimate Partner Violence.

### Social Goals and General Measures of Antisocial Behavior

**Figure 1.**
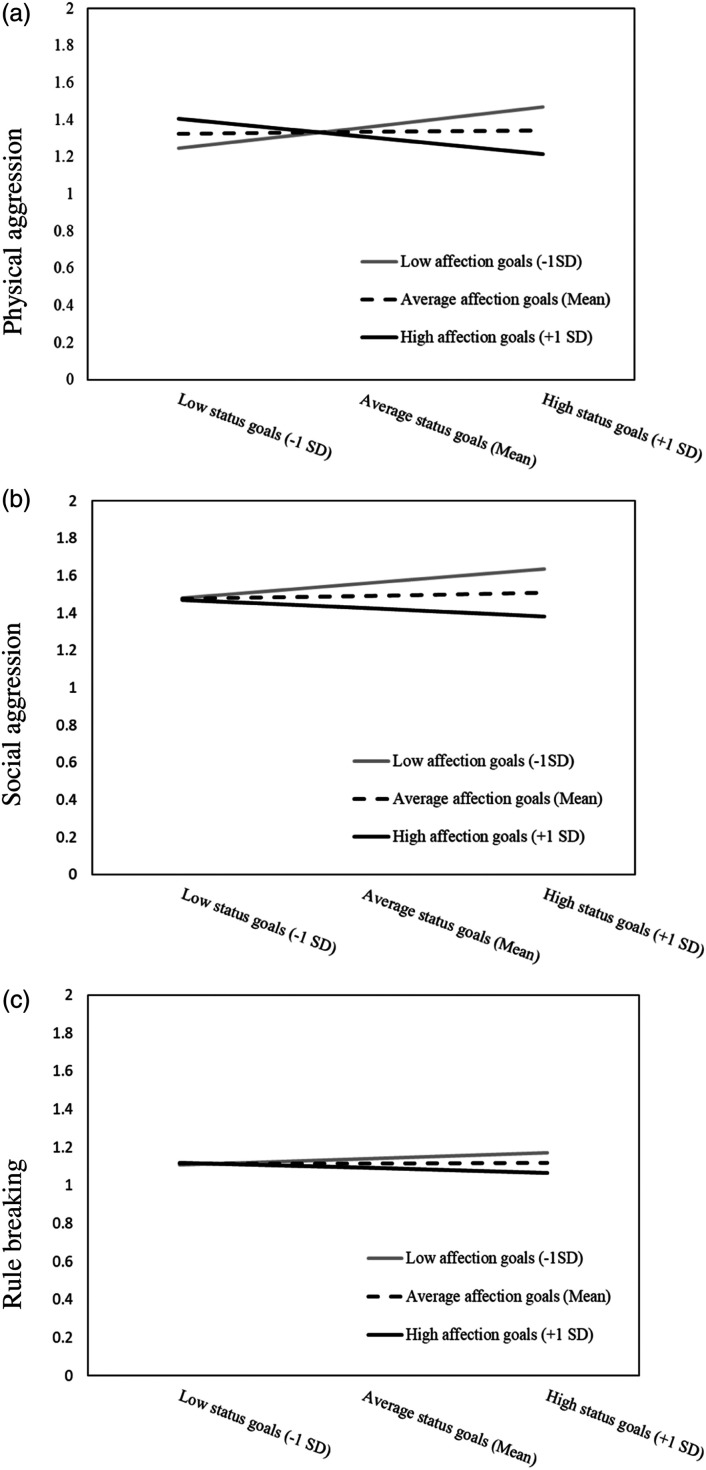
Plots of simple slopes of the association between the status goal and aggression at high (+1 SD), average (Mean), and low (−1 SD) levels of the affection goal for physical aggression (a), social aggression (b), and rule breaking (c).

In Table 3, main and interaction effects of status and affection goals on physical aggression are presented, while accounting for sex and age group. In line with our *Physical aggression hypothesis*, this model showed a significant interaction effect between status goals and affection goals in the explanation of physical aggression (see Figure 1a). Simple slope analyses indicated that the status goal was positively associated with physical aggression, but indeed only at low levels of the affection goal (*b* = 0.11, SE = 0.03, *p* < .001, 95% CI = 0.05 to 0.18). Moreover, a weak status goal was associated with reporting less physical aggression, but only at when the affection goal was strong (*b* = −0.09, SE = 0.03, *p* < .01, 95% CI = −0.15 to −0.04). The Johnson-Neyman significance region suggested that the interaction between status and affection goals became significant at affection goal values of −.39 and lower and .54 and higher, which together comprised 57.7% of the sample.

**Table 3.** *Regression Analyses of Status and Affection Goals on Subtypes of Antisocial Behavior*.

	Physical Aggression				Social Aggression				Rule-breaking			
	B	*SE*	LB	UB	B	*SE*	LB	UB	B	*SE*	LB	UB
Main effects											
Constant	**1.51**	*0.14*	1.23	1.79	**1.60**	.10	1.40	1.79	**1.16**	0.05	1.07	1.25
Sex (1 = male; 2 = female)	−0.07	*0.07*	−0.21	0.07	−0.02	0.05	−0.12	0.09	−0.01	0.03	−0.06	0.04
Age dummy (0 = age 18–30; 1 = age >30)	−**0.20**	*0.04*	−0.27	−0.11	−**0.30**	0.05	−0.40	−0.20	−**0.08**	0.02	−0.13	−0.03
Status goals	−0.00	*0.03*	−0.06	0.06	0.01	0.02	−0.04	0.05	0.00	0.01	−0.02	0.02
Affection goals	−0.01	*0.04*	−0.09	0.07	−**0.06**	0.02	−0.10	−0.02	−**0.02**	0.01	−0.04	-0.00
R^2^	5.9%					14.6%					6.2%	
Two-way interactions											
Constant	**1.41**	*0.10*	1.21	1.61	**1.54**	0.10	1.35	1.74	**1.13**	.05	1.04	1.22
Sex (1 = male; 2 = female)	−0.02	*0.06*	−0.12	0.09	0.01	0.05	−0.09	0.12	0.00	0.03	−0.05	0.05
Age dummy (0 = age 18–30; 1 = age >30)	−**0.19**	*0.05*	−0.29	−0.09	−**0.30**	0.05	−0.40	−0.20	−**0.08**	0.02	−0.12	−0.03
Status goals	0.01	*0.02*	−0.04	0.05	0.02	0.02	−0.03	0.06	0.00	0.01	−0.02	0.02
Affection goals	−0.02	*0.02*	−0.07	0.02	−**0.07**	0.02	−0.11	−0.02	−**0.03**	0.01	−0.05	−0.01
Status goals x affection goals	−**0.11**	*0.02*	−0.15	−0.06	−**0.06**	0.02	−0.10	−0.02	−**0.03**	0.01	−0.05	−0.01
R^2^	14.3%				17.5%				9.6%			

*Note.* Figures in bold face are statistically significant.

Next, we examined the test of our application of the *imbalanced needs theory of aggression* to other forms of aggression. In line with our *Social aggression hypothesis*, effects were similar to those of physical aggression (see Figure 1b): a strong status goal was only positively associated with physical aggression at low levels of the affection goal (*b* = 0.08, SE = 0.03, *p* < .05, 95% CI = 0.01 to 0.14). The Johnson-Neyman significance region suggested that this interaction became significant at affection goal values of −0.58 or lower, which comprised 23.3% of the sample.

In contrast to our *Rule-breaking hypothesis*, where we argued that status goals would be unrelated to rule breaking, we found a significant interaction between status and affection goals. That is, the strength of the status goal was positively associated with rule breaking, but only at low levels of the affection goal (*b* = 0.03, SE = 0.01, *p* < .05, 95% CI = 0.00–0.06) (see Figure 1c). The Johnson-Neyman significance region suggested that this interaction became significant at affection goal values of −.80 and lower, comprising 17.8% of the sample. In this sense, the link between social goals and rule-breaking worked similar to physical and social aggression.

### Social Goals and Aggression in Different Contexts

Next, we tested our hypotheses related to the application of our theory to different contexts, by studying links between the status goal and aggression at the workplace and in romantic relationships. Analogous to the previous analyses, main and interaction effects of status and affection goals are presented, while accounting for sex and age group (see Table 4). Our *Work-setting hypothesis* stated that in the work place, the strength of status goals is only weakly related or unrelated to aggression. This hypothesis (which is also the null hypothesis) was supported: The status goal was unrelated to aggression in the work context. Moreover, the analyses indicated that the status goal did not interact with the affection goal in explaining workplace aggression, but a weaker affection goal was associated with more workplace aggression (*b* = −0.03, SE = 0.02, *p* < .05, 95% CI = −0.07 to 0.00). Thus, in line with our expectation and the null hypothesis, we found no support for a link between status goals and aggression in the workplace.

**Table 4.** *Regression Analyses of Status and Affection Goals on Workplace Violence and Intimate Partner Violence*.

	Workplace Aggression				Negotiation IPV				Psychological IPV			
	B	*SE*	LB	UB	B	*SE*	LB	UB	B	*SE*	LB	UB
Main effects											
Constant	**1.35**	*0.08*	1.20	1.50	**4.07**	*0.49*	3.11	5.04	**0.96**	*0.32*	0.33	1.59
Sex (1 = male; 2 = female)	−0.05	*0.04*	−0.13	0.04	−0.14	*0.27*	−0.67	0.39	0.06	*0.17*	−0.28	0.40
Age dummy (0 = age 18–30; 1 = age >30)	−0.02	*0.04*	−0.10	0.06	−**0.82**	*0.26*	−1.32	−.31	−**0.54**	*0.17*	−0.87	−0.21
Status goals	0.04	*0.02*	−0.01	0.06	0.08	*0.11*	−0.14	0.30	0.05	*0.07*	−0.10	0.19
Affection goals	−**0.03**	*0.02*	−0.07	0.00	0.03	*0.11*	−0.19	0.25	−0.12	*0.07*	−0.26	0.03
R^2^	2.9%				5.0%				6.2%			
Two-way interactions											
Constant	**1.32**	*0.08*	1.17	1.48	**4.03**	*0.50*	3.04	5.01	**0.99**	*0.32*	0.35	1.62
Sex (1 = male; 2 = female)	−0.03	*0.04*	−0.12	0.05	−0.11	*0.27*	−0.65	0.42	0.05	*0.18*	−0.30	0.40
Age dummy (0 = age 18–30; 1 = age >30)	−0.02	*0.04*	−0.10	0.06	−**0.81**	*0.26*	−1.32	−0.31	−**0.54**	*0.17*	−0.87	−0.22
Status goals	0.03	*0.02*	−0.01	0.07	0.09	*0.11*	−0.14	0.31	0.04	*0.07*	−0.10	0.19
Affection goals	−**0.04**	*0.02*	−0.07	0.00	0.02	*0.11*	−0.20	0.24	−0.11	*0.07*	−0.26	0.03
Status goals x affection goals	−0.03	*0.02*	−0.06	0.00	−0.06	*0.10*	−0.26	0.15	0.03	*0.07*	−0.10	0.16
∆R^2^	4.1%				5.2%				6.4%			

*Note.* Figures in bold face are statistically significant.

*Note.* IPV = Intimate Partner Violence.

Finally, we tested our *Romantic context hypothesis* suggesting that the strength of the status goal is not associated with intimate partner aggression, irrespective of the affection goal. In Table 4, we present findings for psychological aggression and conflict negotiation strategies. In line with the hypothesis, main effects indicate that both status and affection goals were unrelated to psychological aggression as well as to conflict negotiation strategies. Moreover, the affection goal did not moderate the association between the status goal and these two types of aggression. Only age group was significantly associated with aggression, suggesting that younger participants were more likely to report the use of psychological aggression and conflict negotiation strategies in their romantic relationships.

## Discussion

In the current study, we provided a further test of the *imbalanced needs theory of aggression*. The theory states that to study the social determinants of aggression one has to look at its link to the satisfaction of fundamental needs (status and affection). Aggression can be used to realize status, but at a cost for realizing affection in the same social context, which would create an imbalance in need satisfaction. The theory implies that social contexts in which status need fulfillments have to be balanced with affection need fulfillment, would be associated with low levels of aggression, except when affection needs are low (that is, when the pursuit of status via aggression cannot create an imbalance). Findings for adolescents (Sijtsema et al., 2020) supported this theory. The question for the present study was whether it would also hold for adults. We conducted five tests of the theory in adult populations. First, we looked at direct aggression (not context specific), and found our prediction supported by the data that the strength of the status goal is only related to aggression when the affection goal is weak. Then we looked at different forms of aggression: social aggression and rule-breaking behavior. We hypothesized that we would find similar results for social aggression, but not for rule breaking, because rule-breaking for the achievement of status is especially prominent in adolescence, not adulthood. We found that status goals were related to both increased social aggression and rule breaking when affection goals were weak. Seemingly, not just social aggression, but also rule-breaking has a negative influence on affection, creating an imbalance even in adulthood. The latter finding goes against our hypothesis. A possible explanation is that, contrary to adolescence, rule-breaking behaviors in adulthood may actually relate to acquiring *means* for status (such as stealing money or selling drugs), which may negatively affect others within the same social circle (e.g., by being put at risk, or by wishing to dissociate from such behavior), leading to a loss in affection for the perpetrator. Thus, to the degree that rule-breaking for status occurs in adulthood, it may be more likely in those with a strong status goal and a relatively weak affection goal.

Finally, we tested the theory for two different contexts: the workplace and romantic relationships. There, we tested an implication of the imbalance need theory of aggression with regard to contexts in which the use of aggression for the achievement of status would *not *occur: contexts in which status can be achieved more easily without aggression (like the workplace), and contexts in which status striving plays a subordinate role in comparison to the striving for affection (as in romantic relationships). These expectations were supported by our results, showing that both in work and romantic settings, the status goal was unrelated to aggression. This also resonates with previous literature showing that at the workplace there are more alternative ways of achieving status (e.g., Cheng et al., 2013; Maner, 2017) and that violence in intimate relationships is often associated with individual traits, such as personality pathology (Holtzworth-Munroe, 2000; Ross & Babcock, 2009).

Overall, the important message of our findings is that for the study of the social determinants of aggression, it is important to consider the possibilities people have to balance need satisfaction of status and affection. When the social circles overlap for the satisfaction of affection and status, the cost of losing affection by achieving status via aggression is high, rendering this social source of aggression less likely. Similarly, there are social contexts that elevate affection needs far above status needs (such as romantic relationships).

### Scientific and Societal Implications

For the study of aggression, this implies a recommendation for special attention to the ways these two social needs are and can be satisfied, and the relative social importance of these needs. To the degree that the circles overlap or the context elevates affection needs far above status needs, it becomes more likely that aggression derives not from social circumstances but from personality pathology and related factors, such as negative emotions and poor self-control (Douglas & Martinko, 2001; Sotelo & Babcock, 2013), or particularly low levels of affection needs (e.g., as seen in relation to psychopathy: Moreira et al., 2014). Conversely, social contexts that separate the circles of status and affection (such as strong power differences between social groups or the public arena in which the anonymity and lack of past and future social encounters makes status striving with aggression likely), are likely to foster social sources of aggression. Similarly, social contexts that elevate status needs far above affection needs (such as highly competitive contexts) are likely to create social sources of aggression. In such cases, aggression may be triggered by cues from the environment (e.g., work stress or power differences), if aggressive behavior is possible (i.e., low costs to the use of aggression) and the outcome could also be beneficial to the aggressor (cf. Salin, 2003). As such, this theory would not only explain variation in proactive forms of aggression, but also in reactive forms of aggression. These recommendations for further scientific study also offer avenues for policy as it can influence both the overlap of social circles and the relative importance of status goals in comparison to affection goals, thereby influencing the social determinants of aggression.

### Limitations

Despite the importance of these findings, the current study is limited in several respects. First, although it was a great advantage that status and affection goals were measured directly, self-reports also suffer from reporter biases and social desirability. Second, in terms of the diversity of our sample, it should be noted that women were over-represented, which may have affected our findings. In particular, several studies have shown that women display less physical aggression as compared to men (Hyde, 2005). However, in our study, we mainly included social and psychological aggressive behaviors, which have been found to be equally frequent in males and females (Little et al., 2003; Heilbron & Prinstein, 2008). Moreover, we showed that the pattern of findings for general indices of aggression is similar for both direct and more covert forms of aggression. Finally, a convenience sample was collected using a snowball-technique, which may have led to a more homogeneous sample as participants may have come from the same social group or the same area. We should thus be careful in generalizing the current findings to other populations that may show more variation in gender, socio-economic background, or culture. Also, some caution is warranted because the effects that we found were by and large modest, thus suggesting that additional factors, such as personality and social influences not considered by us, are important to include when explaining variations in self-reported aggression.

Notwithstanding these shortcomings, we provided an extensive test of the imbalanced needs theory of aggression, testing its merits in different contexts and pertaining to different forms of antisocial behavior. It stands to reason that replication and further extension of the current study is needed. Avenues that are worth exploring include a more thorough analysis of the conditions under which the satisfaction of status and affection needs becomes imbalanced. For example, under what conditions do power differences in organizations create such an imbalance, and when does competition in organizations create an imbalance between the importance of status and affection goals? Also, neighborhood studies could profit from looking into the degree to which ethnic heterogeneity creates separate social circles for status and affection need satisfaction. There is potentially a wide application of the imbalanced needs theory of aggression in research on the social determinants of aggression.
